# Estimating Human Cases of Avian Influenza A(H7N9) from Poultry Exposure

**DOI:** 10.1371/currents.outbreaks.264e737b489bef383fbcbaba60daf928

**Published:** 2013-05-15

**Authors:** Caitlin Rivers, Kristian Lum, Bryan Lewis, Stephen Eubank

**Affiliations:** Network Dynamics and Simulation Science Laboratory, Virginia Bioinformatics Institute, Virginia Tech, Blacksburg, VA, USA; Network Dynamics and Simulation Science Laboratory, Virginia Bioinformatics Institute, Virginia Tech, Blacksburg, VA USA; Network Dynamics and Simulation Science Laboratory, Virginia Bioinformatics Institute, Virginia Tech, Blacksburg, VA USA; Network Dynamics and Simulation Science Laboratory, Virginia Bioinformatics Institute, Virginia Tech, Blacksburg, VA USA

## Abstract

In March 2013 an outbreak of avian influenza A(H7N9) was first recognized in China. To date there have been 130 cases in human, 47% of which are in men over the age of 55.The influenza strain is a novel subtype not seen before in humans; little is known about zoonotic transmission of the virus, but it is hypothesized that contact with poultry in live bird markets may be a source of exposure. The purpose of this study is to estimate the transmissibility of the virus from poultry to humans by estimating the amount of time shoppers, farmers, and live bird market retailers spend exposed to poultry each day. Results suggest that increased risk among older men is not due to greater exposure time at live bird markets.

## Background

On April 1 the first cases of avian influenza A(H7N9) in Chinese patients who became severely ill with an influenza-like illness were reported to the WHO^1^. Chinese health officials determined that a novel influenza was the source of the illness. Additional cases were soon diagnosed in other regions, including Beijing which is geographically distant. To date there have been 130 cases in eight provinces and two municipalities in China, and new cases continue to be diagnosed^2^. The novel influenza has not yet been found in any other countries, with the exception of a case imported to Taiwan from mainland China^3^.

Virologists determined that the virus was an avian subtype, and that poultry were a possible reservoir^4^. Since many of the first human infections were found in people that had been exposed to poultry, epidemiologists hypothesized that the virus was transmitted to humans after close contact with birds^5^^,^ 6. On April 5, 2013, Shanghai authorities ordered the closing of the city’s live bird markets, which are food markets where live poultry are slaughtered and sold^7^. These markets were suspected sources of exposure for the novel influenza, and their closing was meant to minimize human exposure to infected birds. The WHO notes that poultry as a source of exposure is still unconfirmed^1^.

Among the 130 confirmed cases, 88 (68%) are men, 37 (28%) are women, and 5 (4%) are children. The mean age of the infected is 62 for men, and 61 for women; the median ages are 60.5 and 58, respectively. The age distribution of the influenza A(H7N9) trends much older than previous outbreaks; influenza A(H5N1) affected primarily people ages 20-30, and did not affect men more than women^1^^,^ 8. Thirty-one (24%) of the infected have died; of the 24 deaths for whom demographic data are available, 18 are men (75%). It is still unknown why men are more affected than women, but the WHO suggests that patterns of exposure to live bird market may put certain demographic groups into more contact with the virus^1^.

## Methods

All source data and analyses are available on Figshare (see Appendix).

A line listing of the 130 confirmed cases was developed using data from a variety of sources, including the World Health Organization (WHO)^9^, HealthMap^10^, and Flutrackers.com^11^. Data on the patients’ demographics, course of the disease, and possible exposures were aggregated from media reports and public health organization updates to form as complete of a case record as possible. The patients’ age, sex, and date of illness onset were used as unique identifiers to prevent duplication and data errors. Only cases with a reported onset date between March 13, 2013 and April 11, 2013 (7 days after the live bird markets were closed, assumed to be the incubation period) were used in the analysis. Cases with no available onset date that were announced before April 22, 2013 were included. Cases found in provinces other than Shanghai, Anhui, Zhejiang and Jiangsu, where a majority of cases were found during the analysis period, were also excluded, as were children under the age of 15. The final case count used in analysis is 83.

Data for estimating exposure time to poultry were collected from the National Bureau of Statistics of China. Where possible, indicators were limited to the four analysis provinces and municipalities (Shanghai, Anhui, Zhejiang, Jiangsu). The population for each of the four regions was estimated from a 2005 survey of 1% of Chinese residents, stratified by age group and sex^12^. An estimate of the number of people who visit a live bird market each day was derived from a 2008 time use survey of the proportion of the population that participated in the purchase of goods and services, also stratified by age group and sex^13^. Data were not available for people over the age of 75, so values from the 65-74 age group were applied.

Population counts and the proportion that shop each day were multiplied to estimate the number of people who shop daily. Various sources estimate that about 80% of Chinese residents shop at a market where live poultry are more likely to be present, rather than a supermarket^14^^,^ 15. The reported analysis uses a uniform distribution of 80% live bird market attendance.

The time use survey also provided the number of minutes each day people spend shopping for goods and services^16^. A native Chinese colleague estimated that a trip to the wet market takes approximately 15-30 minutes, which is about half what the time use survey estimates is spent on purchasing goods and services. The time use values were therefore halved to estimate the number of minutes that are spent specifically at the live bird market, rather than shopping for other things.

The number of exposure days was determined to be 30, derived from seven days (assumed to be the incubation period) before the outbreak began to take hold on March 13 until the markets closed on April 5. The number of people exposed at a live bird market each day was multiplied by the number of minutes spent shopping and by the number of exposure days in order to estimate the group exposure hours per day.

A similar procedure was followed to obtain the occupational exposure rate for agriculture workers and live bird market retailers in each of the four affected regions. The number of men and women in “farming, forestry, animal husbandry and fishing” and “wholesale and retail trade” occupations were taken from 2006 labor statistics provided by the National Bureau of Statistics of China^17^. There are an estimated 9.48 million poultry farming jobs in China, which is 15% of the number of workers in the occupational category as a whole^18^. This multiplier was used to narrow down the number of occupational workers exposed to poultry specifically. Daily exposure minutes were derived from labor statistics on weekly working hours for both occupations^19^. The estimated population exposed was then multiplied by the number of exposure minutes per day and the 30 exposure days to derive occupational group exposure hours.

For each case in the line listing, an exposure category was determined where possible. Seven cases were determined to be agricultural workers, and another four were retail (including food preparation) workers. The remaining 72 cases had no information about possible sources of exposure; these were presumed to be transient exposures, and were therefore categorized as shoppers. This estimate is supported by a Morbidity and Mortality Weekly Report that found 77% of confirmed H7N9 cases were exposed to live animals, "primarily chickens (76%) and ducks (20%)"^2^. An infection rate per hour of contact was estimated by dividing the number of cases for each demographic and exposure category by the group exposure hours.

The rate of infection per exposure hour for demographic group \begin{equation*}i\end{equation*} is given by the equation:

\begin{equation*}rate_i = \frac{cases_i}{population_i\:\times proportion\:who\:visit\:LBM_i\times exposure\:time_i}\end{equation*}

## Results and discussion

**Human infection rate per exposure hour to poultry:**
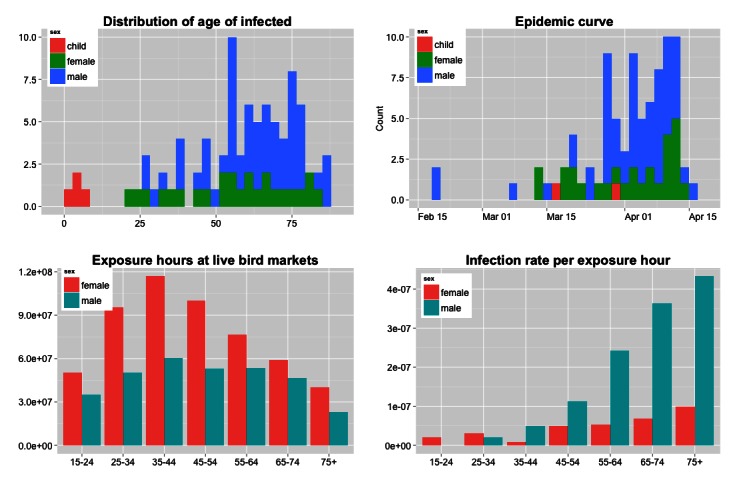
Men ages 55+ are disproportionately affected by avian influenza A(H7N9). Despite having a lower estimated exposure time to live bird markets, older men have a much higher infection rate per exposure hour than other demographic groups.

Among shoppers, estimated infection rates per hour increase with age, particularly among men. For example, the infection rate for men ages 65-74 is over five times as high as it is for women of the same age, and over eighteen times as high as for men ages 25-34 (see figure 1, bottom right panel). Shoppers under the age of 35 have very few infections (n=5 adults, n=4 children), despite having in some cases greater exposure hours than older demographics. Although the time use survey shows that older people spend more time shopping than younger people, there are fewer older people in the population overall. Among both men and women shoppers, the group exposure hours (the population at risk multiplied by the time spent exposed) are highest for those ages 35-44 (see figure 1, bottom left panel).

Infection rates among occupationally-exposed people were estimated to be lower than among shoppers, likely because the amount of time spent in contact with poultry was overestimated. No data were available to refine these estimations. Nonetheless, the pattern remains the same in that occupationally-exposed men have a higher infection rate than occupationally-exposed women.

A sensitivity analysis shows that these conclusions are quite robust. As noted, an estimated 80% of Chinese residents attend live bird markets^14^^,^ 15. However, attendance rates within age and sex groups are unknown. To investigate whether it is possible that the lower number of cases for younger people is due to lower live bird market attendance rather than a decreased transmission rate for those age groups, we decreased bird market attendance rates for younger people to the extent possible while maintaining a marginal attendance rate of 80%. Attendance rates under this scenario are shown in the middle panel of Figure 2. Even under these extreme assumptions, the infection rate per exposure hour still strongly favors older men (see bottom panel of figure 2). In fact, in order for the infection rate for all age and sex groups to have been the same as that of the highest (assuming 100% live bird market attendance for the highest group), attendance rates for all other groups would have to be between 2-30%, with an average population-wide attendance rate of about 18%. This is inconsistent with the 80% figure reported. These findings are also supported by a study of the annual number of poultry exposures from live bird market visits and backyard poultry in four areas of China^8^. The poultry exposure data reported by Cowling et al yield very similar infection rates per exposure hours as is reported here. More details are available in Appendix 2.

**Sensitivity analysis:**
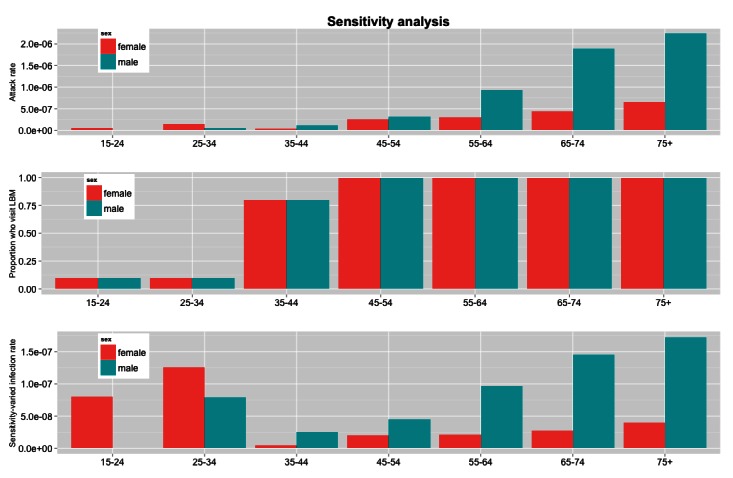
The hypothetical number of undetected cases can be estimated assuming that the infection rate per exposure hour is constant, using men ages 75+ as a reference group.

The present methodology can also be used for hypothesis-generating analyses. If we suppose that the infection rate per exposure hour is in fact uniform across all groups, and that men ages 75+ (for whom attack rates are the highest) have perfect case detection in which all infections are identified, we can estimate the percentage of undetected cases in other demographics. Under those assumptions, women ages 35-44 would have the lowest detection rate at around 2%, meaning that 98% of cases are not detected (see figure 3). These results suggest that biases in case detection are not the sole cause of the age and sex distribution seen in this outbreak.

**Hypothetical detection rate using men ages 75+ as the reference group:**
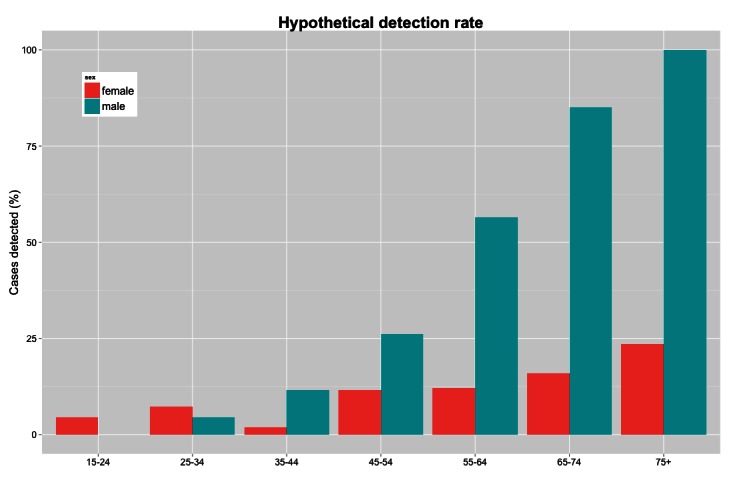
The hypothetical number of undetected cases can be estimated assuming that the infection rate per exposure hour is constant, using men ages 75+ as a reference group.

## Conclusion

We have shown that the infection rates per exposure hour for avian influenza A(H7N9) are likely much higher among older populations, particularly men. It is implausible that these discrepancies are due to differences in the rates of market attendance or systematic under-reporting. These findings suggest that the age distribution of the outbreak is due to an as-yet unknown epidemiological or immunological feature, and is not due to greater exposure to poultry among the older demographics. It should be noted that for many of the demographic groups, the case counts are quite low, resulting in instability in the rate calculations. It should also be noted that assumptions about the live bird market attendance and duration, and the incubation period, may affect results. That said, the overall conclusions have proven robust to changes in assumptions. Clearly, additional data is needed to confirm the existence of the striking differences in transmission rate by demographic group. Even without extensive epidemiological data, this analysis provides timely evidence that other factors may be contributing to the differential case detection of older men than simply market exposure, as has been hypothesized by the WHO and others.

## Figures and Tables

**Comparison of exposure estimates reported by Cowling et al:**
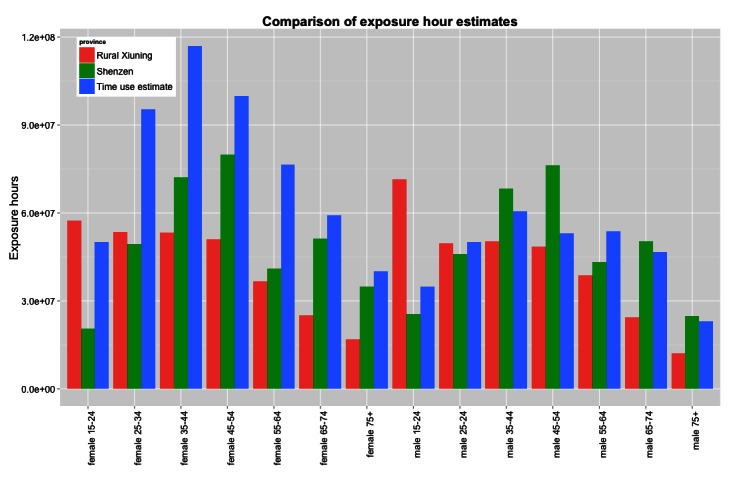
Cowling et al reported on the number of annual exposures to poultry in live bird markets and backyards in four regions of China^8^. The survey was conducted in regions not reported on here, and findings were not stratified by sex. The provinces with the highest and lowest exposures were chosen to represent upper and lower bounds. Exposure values were scaled to match the time period of 30 days and applied to both male and female exposure categories. The resulting group exposure hours and infection rate per exposure hours are similar to those reported here.

## Appendix 1

### Data availability

All source data and analyses are freely available for download on Figshare here (http://dx.doi.org/10.6084/m9.figshare.688050).
